# Distribution of Endosymbiotic Reproductive Manipulators Reflects Invasion Process and Not Reproductive System Polymorphism in the Little Fire Ant *Wasmannia auropunctata*

**DOI:** 10.1371/journal.pone.0058467

**Published:** 2013-03-11

**Authors:** Olivier Rey, Arnaud Estoup, Benoit Facon, Anne Loiseau, Alexandre Aebi, Olivier Duron, Fabrice Vavre, Julien Foucaud

**Affiliations:** 1 CBGP UMR1062, INRA, Montpellier, France; 2 Laboratoire de Biologie du Sol, Université de Neuchâtel, Neuchâtel, Switzerland; 3 Institut des Sciences de l’Evolution UMR5554, CNRS, Université Montpellier 2, Montpellier, France; 4 Laboratoire de Biometrie et Biologie Evolutive UMR5558, CNRS, Université Lyon 1, Villeurbanne, France; University of Poitiers, France

## Abstract

Endosymbiotic reproductive manipulators may have drastic effects on the ecological and evolutionary dynamics of their hosts. The prevalence of these endosymbionts reflects both their ability to manipulate their hosts and the history of the host populations. The little fire ant *Wasmannia auropunctata* displays a polymorphism in both its reproductive system (sexual versus clonal populations) and the invasive status of its populations (associated to a habitat shift). We first screened for the presence of a diverse array of reproductive parasites in sexual and clonal populations of *W. auropunctata*, as a means to investigate the role of endosymbionts in reproductive phenotypes. *Wolbachia* was the only symbiont found and we then focused on its worldwide distribution and diversity in natural populations of *W. auropunctata*. Using a multilocus scheme, we further characterized the *Wolbachia* strains present in these populations. We found that almost all the native sexual populations and only a few clonal populations are infected by *Wolbachia*. The presence of similar *Wolbachia* strains in both sexual and clonal populations indicates that they are probably not the cause of the reproductive system polymorphism. The observed pattern seems rather associated to the invasion process of *W. auropunctata*. In particular, the observed loss of *Wolbachia* in clonal populations, that recurrently emerged from sexual populations, likely resulted from natural heat treatment and/or relaxed selection during the shift in habitat associated to the invasion process.

## Introduction

Maternally inherited symbionts are extremely common in arthropods and constitute an important force in evolution, because their transmission success is tightly linked to host fitness. These symbionts may have drastic effects on the ecological and evolutionary dynamics of their hosts [1]–[3]. Infections can both results in fitness costs [4]–[5] and/or benefits, such as defense against natural enemies [6]–[9]. The fitness cost-benefit ratio, eventually driving the prevalence of endosymbionts in natural host populations, is likely to be influenced by environmental parameters, such as abiotic conditions [10] or parasitism pressure [11]–[12]. In habitats where interspecific interactions are frequent and parasitism elevated, the benefits of an endosymbiotic-driven defense may lead to high infection prevalence, while in habitats where parasite pressure is low, incurred costs may purge populations from infection [12]–[13]. This process can have important consequences in the context of an exotic habitat change (e.g. during an introduction event), in which introduced populations are likely to face different, sometimes lower, parasite pressures (i.e., the enemy release hypothesis; [14]). In particular, in the case of a trade-off between immunological and demographic traits (e.g., defensive endosymbiotic bacteria may reduce fecundity), purge of endosymbiotic bacteria following an enemy release is expected to promote the invasion process [15]–[17]. Accordingly, loss of parasites is frequent in a variety of invasive species [16–18–20]. It is thus important to investigate possible gains or losses of endosymbiotic bacteria and their effects in the context of invasive events [16].

Apart from general effects over fitness, some maternally inherited symbionts are also able to manipulate the reproduction of their host [21]–[22]. These latter parasites alter the reproductive system of their host so that the number of infected daughters produced by an infected female exceeds the average production of daughters per uninfected female. Studies that have investigated reproductive parasitism have long been biased towards *Wolbachia*. This is logical, to some extent, given that it is the most widespread endosymbiotic bacterium within arthropods [23]. To date, this bacterium has been found to induce four manipulation phenotypes: cytoplasmic incompatibility, feminization of genetic males, male killing and induction of thelytokous parthenogenesis [22]–[24]. In those cases, the haplodiploid genetic system seems to be a strong predisposition [22]. *Wolbachia* is, however, just one of the known reproductive parasites, and other endosymbiotic bacteria are now receiving increased attention [22–25–26]. *Cardinium* has been shown to induce cytoplasmic incompatibility, feminization and induction of parthenogenesis, *Rickettsia* can cause parthenogenesis and male killing and finally *Arsenophonus* as well as *Spiroplasma* can induce male killing within their hosts. All of these reproductive parasites lead to biases in reproductive schemes, which are beneficial for infected matrilines and, therefore, may have drastic effects on the evolutionary dynamics of their hosts [21]–[22]. It is therefore crucial to investigate their presence and their potential roles in organisms that present peculiar reproductive systems.

In the little fire ant, *Wasmannia auropunctata*, a small myrmicine species originating from South America and introduced worldwide, infection pattern of endosymbiotic bacteria could reflect both the invasion process and/or a reproductive manipulation. Indeed, this species displays polymorphisms both in the invasive status of its populations (associated to a habitat shift; [27]–[28]), and reproductive system (sexual vs. clonal populations; [29]–[30]). Within its native range, one can distinguish two types of *W. auropunctata* populations regarding invasive status. First, ancestral populations are confined within the primary forest and are characterized by low nest and worker densities [27]–[28]. Second, more recent invasive populations repeatedly colonized human modified habitats within the native range (e.g. road sides, plantations; [28]). This change of habitat is associated with a major ecological shift, with high worker and nest densities [28]. By contrast, only the invasive type of populations (high worker and nest densities) is found within the introduced range, in human modified habitats [27]. Similarly to other invasive ant species [18]–[20], one could expect that the habitat shift of invasive populations within and outside the native range of *W. auropunctata* is associated with the loss of their endosymbiotic bacteria.

Apart from the possible effects on host fitness, endosymbiotic bacteria could also play a role in the peculiar reproductive system polymorphism displayed by *W. auropunctata* [29]–[30]. In some populations (hereafter called “sexual populations”), queens and males reproduce following a classical haplo-diploid scheme where diploid females (i.e. queens and sterile workers) are produced sexually and haploid males develop from haploid eggs through arrhenotoky. Some populations (hereafter called “clonal populations”) emerged recurrently from these sexual populations, in which reproductives (i.e. queens and males) display an uncommon reproductive system: queens use thelytokous parthenogenesis and sexual reproduction in a conditional manner to produce gynes (i.e. unfertilized queens) and (sterile) workers, respectively [29]–[30]. A recent study revealed that gynes are produced via automictic parthenogenesis with central fusion [31]. Moreover, contrary to other species in which queens reproduce by thelytoky, unmated queens are unable to lay viable eggs [29]. The production of parthenogenetic eggs seems therefore strictly dependent on the fertilization process. Finally, female parthenogenesis is tightly associated to male clonality [29]–[30]. The genome of males is transmitted clonally via maternal eggs through a mechanism yet unresolved. Interestingly, a similar reproductive system was described in two unrelated ant species, *Vollenhovia emeryi* [32] and *Paratrechina longicornis* [33]. Current knowledge in Hymenoptera does, however, not plead for the involvement of endosymbiotic manipulators in this reproductive system. Indeed, in all cases of symbiont-induced parthenogenesis examined so far, restoration of diploidy is achieved through a process of gamete duplication, making daughters completely homozygous and precluding this kind of effect in species exhibiting complementary sex determination, such as ants. However, other mechanisms may exist, as in two mite species where *Wolbachia*-induced parthenogenesis is achieved through a mechanism that is functionally apomictic, as daughters keep the heterozygosity of their mothers [34]. While some evidence would suggest that this peculiar reproductive system might be under genetic determinism [30], no cause has been demonstrated so far, and endosymbiotic manipulation still stands as a possible explanation.

Interestingly, the reproductive system polymorphism is almost always associated to the invasive status of populations [27]–[35]. While native non-invasive populations are mainly sexual, both native and introduced invasive populations are clonal. This leads to opposite expectations regarding the pattern of endosymbiontic bacterial infection in populations of *W. auropunctata*, under the hypotheses of an endosymbiotic role in either the invasion process or the reproductive system determinism. If endosymbionts are involved in the determinism of the reproductive system, then the infection pattern would display an excess of infection in clonal compared to sexual populations, and/or clonal and sexual populations would not display similar strains of endosymbionts. On the contrary, from an invasion biology perspective, the expectation of infection pattern would be that invasive clonal populations, within and outside the native range, are free of endosymbionts and native sexual populations are infected.

To distinguish between these hypotheses, we conducted an extensive screening of *W. auropunctata* native sexual populations and clonal populations from both the native and the invasive range, for five known reproductive parasites, *Wolbachia*, *Arsenophonus*, *Cardinium*, *Rickettsia* and *Spiroplasma ixodetis*. *Wolbachia* was the only detected endosymbiont and we then analyzed its distribution among worldwide *W. auropunctata* populations from both types (i.e. sexual and clonal) in the native and introduced range to identify major correlates and patterns that could indicate the driving force of infection patterns in this species (i.e. reproductive system versus invasion process). We additionally examined the *Wolbachia* diversity through *W. auropunctata* population by sequencing a combination of six bacterial genes to better refine our understanding of the history of infection in this species.

## Materials and Methods

### Screening for Endosymbiotic Bacteria

**Figure 1:**
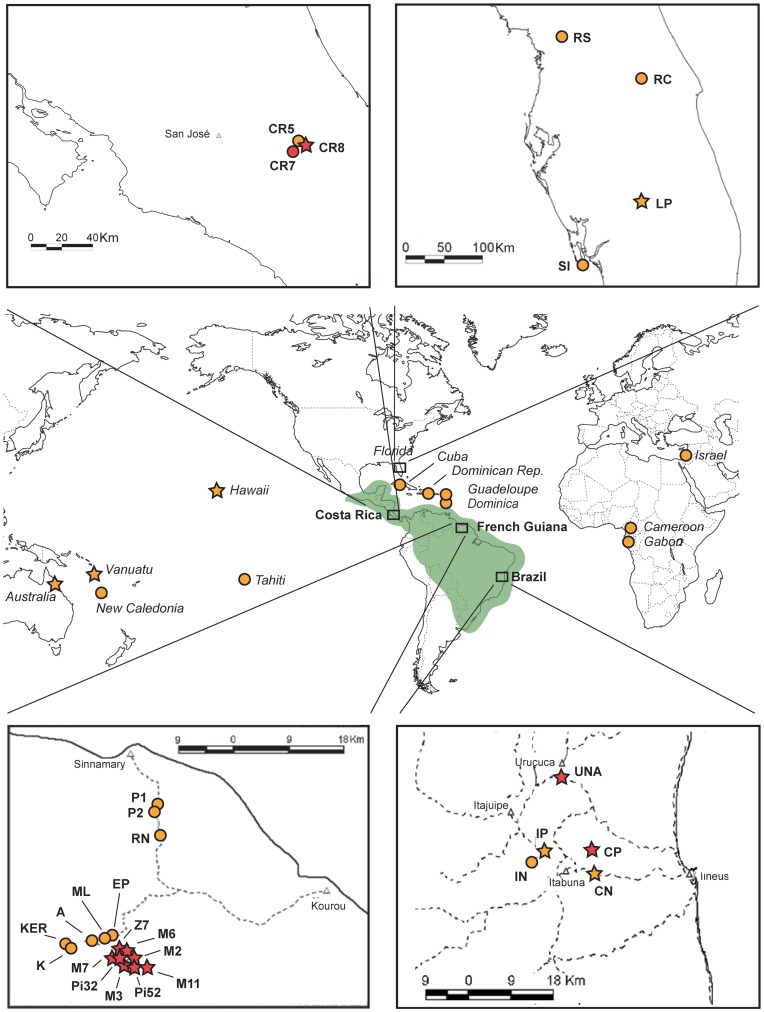
Map of the sites sampled to assess the presence of endosymbionts in *W. auropunctata* populations. Note: The presence of five endosymbionts was investigated at 42 sites worldwide, and *Wolbachia* was investigated at 56 sites (listed in Table 2). The native range of *W. auropunctata* is indicated by green shading. Clonal and sexual populations are indicated in orange and red, respectively. *Wolbachia*-infected and non-infected populations are indicated with stars and circles, respectively. When all populations of a given country displayed the same infection status and reproductive system, a single point was added to the map. On the contrary, all populations from a single country are indicated in a dedicated window when populations showed a polymorphism in infection status and/or reproductive system, i.e. for all native (Brazil, Costa Rica, French Guiana) and one introduced populations (Florida).

We used specific PCR-amplification to investigate for the presence of *Wolbachia*, *Cardinium*, *Arsenophonus*, *Rickettsia* and *Spiroplasma ixodetis* on 84 individuals collected in 42 nests from 42 populations (one nest per population) covering most of the distribution of *W. auropunctata*, including its native and introduced range (Figure 1). Sampling was conducted in public locations that did not required specific authorization. In the native range nine sexual and 12 clonal populations were available from a previous study [27]. In the introduced range, 21 clonal populations were available [35]. When possible (35 nests), a queen and a worker from the same nest were screened. In seven nests no queens were sampled and two workers were thus screened for infection.

The total genomic DNA was extracted from all individuals following a standard CTAB protocol. We performed PCR-based screening using a protocol modified from Shoemaker *et al.* [19] for *Wolbachia* detection, and following the protocols described in Duron *et al.* [25] for the four other endosymbionts. To avoid false negatives, we used an arthropod specific DNA fragment of the *W. auropunctata EF1α* gene to serve as an internal control of DNA extractions and PCR quality, using the universal primer set trs4F/trs9R [36]. Positive controls infecting *Culex pipiens (*Diptera: Culicidae), *Holocnemus pluchei* (Araneae: Pholcidae), *Hippobosca equina* (Diptera: Hippoboscidae), *Bemisia tabaci* (Hemiptera: Aleyrodidae) and *Cicadella viridis* (Hemiptera: Cicadellidae) were used in each PCR for the screening of *Wolbachia*, *Cardinium*, *Arsenophonus*, *Rickettsia* and *Spiroplasma ixodetis* respectively. Negative controls (i.e. ultrapure water) were used in each PCR. PCR products were electrophoresed in 1.5% agarose gels and visualized under UV illumination after staining in an ethidium bromide solution.

### Wolbachia-specific Additional Screening

Among the five endosymbionts screened, *Wolbachia* was the only symbiont detected in some *W. auropunctata* populations (see Results section). We extended our screening of *Wolbachia* on a larger dataset to more accurately assess the distribution of this endosymbiont among *W. auropunctata* populations. Together with individuals previously examined, a total of 205 queens and 376 workers were screened following the protocol detailed in the previous section. Altogether, these individuals screened for *Wolbachia* infection originated from 174 and 248 nests for queens and workers, respectively, from 56 worldwide populations (Table 1 and 2; Figure 1).

**Table 1:** Prevalence of *Wolbachia* infection in *W. auropunctata* queens from nests sampled in the native and introduced range.

Range	Location	Site	Reproductive system	N(nests)	N(Queens)	*Wolbachia* infection prevalence
Native	French Guiana	M2	Sexual	4	4	1
Native	French Guiana	M3	Sexual	5	5	1
Native	French Guiana	M6	Sexual	2	6	1
Native	French Guiana	M7	Sexual	9	13	1
Native	French Guiana	M11	Sexual	3	3	1
Native	French Guiana	Z7	Sexual	1	1	1
Native	French Guiana	Pi32	Sexual	1	2	1
Native	French Guiana	Pi52	Sexual	1	1	1
Native	Brazil	CP	Sexual	10	10	1
			**Sexual**	**36**	**45**	**1**
Native	French Guiana	K	Clonal	3	8	0
Native	French Guiana	P1	Clonal	3	11	0
Native	French Guiana	P2	Clonal	17	24	0
Native	French Guiana	RN	Clonal	5	5	0
Native	French Guiana	A	Clonal	2	2	0
Native	French Guiana	ML	Clonal	1	1	0
Native	French Guiana	EP	Clonal	1	1	0
Native	Brazil	CN	Clonal	13	13	1
Native	Brazil	IP	Clonal	10	10	1
Native	Brazil	IN	Clonal	5	5	0
Introduced	New Caledonia	BS	Clonal	4	4	0
Introduced	New Caledonia	BT	Clonal	7	8	0
Introduced	New Caledonia	PL	Clonal	4	4	0
Introduced	Tahiti	PF	Clonal	4	4	0
Introduced	Gabon	LO	Clonal	7	7	0
Introduced	Gabon	EK	Clonal	5	5	0
Introduced	Gabon	OK	Clonal	2	2	0
Introduced	Gabon	LBV	Clonal	1	1	0
Introduced	Gabon	NT	Clonal	2	2	0
Introduced	Cameroon	AK	Clonal	4	4	0
Introduced	Cameroon	BD	Clonal	5	5	0
Introduced	Cameroon	BY	Clonal	5	5	0
Introduced	Cameroon	SM	Clonal	4	4	0
Introduced	Israel	ME	Clonal	1	1	0
Introduced	Israel	KI	Clonal	1	1	0
Introduced	Israel	AF	Clonal	1	1	0
Introduced	Israel	BZ	Clonal	1	1	0
Introduced	USA (Florida)	RS	Clonal	1	1	0
Introduced	USA (Florida)	RC	Clonal	1	1	0
Introduced	Hawaii	Ha	Clonal	3	3	1
Introduced	Guadeloupe	FDI	Clonal	1	1	0
Introduced	Guadeloupe	CAE	Clonal	1	1	0
Introduced	Cuba	CU	Clonal	1	2	0
Introduced	Vanuatu	BK	Clonal	5	5	1
Introduced	Vanuatu	SA	Clonal	1	1	1
Introduced	Australia	CA	Clonal	6	6	1
			**Clonal**	**138**	**160**	**0,167**

**Table 2:** Prevalence of *Wolbachia* infection in *W. auropunctata* workers from nests sampled in the native and introduced range.

Range	Location	Site	Reproductive system	N(nests)	N(Workers)	*Wolbachia* infection prevalence
Native	French Guiana	M2	Sexual	8	14	0.93
Native	French Guiana	M3	Sexual	10	18	1
Native	French Guiana	M6	Sexual	6	14	1
Native	French Guiana	M7	Sexual	15	22	0.91
Native	French Guiana	M11	Sexual	10	16	1
Native	French Guiana	Z7	Sexual	6	10	1
Native	French Guiana	Pi32	Sexual	1	8	1
Native	French Guiana	Pi52	Sexual	1	2	1
Native	Brazil	UNA	Sexual	10	20	1
Native	Brazil	CP	Sexual	10	15	1
Native	Costa Rica	CR8	Sexual	1	2	1
Native	Costa Rica	CR7	Sexual	1	1	0
			**Sexual**	**79**	**142**	**0.903**
Native	French Guiana	K	Clonal	3	5	0
Native	French Guiana	P1	Clonal	4	4	0
Native	French Guiana	P2	Clonal	16	20	0
Native	French Guiana	RN	Clonal	6	8	0
Native	French Guiana	Ker	Clonal	2	2	0
Native	French Guiana	A	Clonal	2	2	0
Native	French Guiana	ML	Clonal	2	2	0
Native	French Guiana	EP	Clonal	2	2	0
Native	French Guiana	Cay	Clonal	1	3	0
Native	Brazil	CN	Clonal	13	27	0.78
Native	Brazil	IP	Clonal	10	10	1
Native	Brazil	IN	Clonal	10	15	0
Native	Costa Rica	CR5	Clonal	1	2	0
Introduced	New Caledonia	BS	Clonal	4	4	0
Introduced	New Caledonia	BT	Clonal	8	8	0
Introduced	New Caledonia	PL	Clonal	4	4	0
Introduced	Tahiti	PF	Clonal	4	9	0
Introduced	Gabon	LO	Clonal	7	7	0
Introduced	Gabon	EK	Clonal	6	6	0
Introduced	Gabon	OK	Clonal	3	3	0
Introduced	Gabon	LBV	Clonal	1	1	0
Introduced	Gabon	NT	Clonal	2	2	0
Introduced	Cameroon	AK	Clonal	5	5	0
Introduced	Cameroon	BD	Clonal	5	5	0
Introduced	Cameroon	BY	Clonal	5	5	0
Introduced	Cameroon	SM	Clonal	5	5	0
Introduced	Israel	ME	Clonal	1	1	0
Introduced	Israel	KI	Clonal	1	1	0
Introduced	Israel	AF	Clonal	1	1	0
Introduced	Israel	BZ	Clonal	1	1	0
Introduced	USA (Florida)	RS	Clonal	1	1	0
Introduced	USA (Florida)	RC	Clonal	1	1	0
Introduced	USA (Florida)	LP	Clonal	1	5	1
Introduced	USA (Florida)	SI	Clonal	1	2	0
Introduced	Hawaii	HA	Clonal	3	9	1
Introduced	Guadeloupe	FD	Clonal	7	7	0
Introduced	Guadeloupe	CAE	Clonal	2	2	0
Introduced	Guadeloupe	PDM	Clonal	1	1	0
Introduced	Cuba	CU	Clonal	2	7	0
Introduced	Vanuatu	BK	Clonal	5	10	1
Introduced	Vanuatu	SA	Clonal	1	2	1
Introduced	Australia	CA	Clonal	6	8	1
Introduced	Dominica	CO	Clonal	2	6	0
Introduced	Dom. Rep	DR	Clonal	1	3	0
			**Clonal**	**169**	**234**	**0.154**

Based on this additional screening, we estimated the prevalence of *Wolbachia* within populations in the queen and worker castes using the ratio of the number of infected individuals to the total number of screened individuals of the same caste. To indirectly test whether *Wolbachia* could be involved in the reproductive system polymorphism of *W. auropunctata*, we tested for a statistical association between the reproductive system within nests (i.e. sexual or clonal) and the infection status (i.e. presence or absence of *Wolbachia*) of individuals within the nests. Nests with at least one individual infected were considered as infected, and nests in which all individuals were *Wolbachia*-free were considered as non-infected. The association was tested using a Fisher's exact test and the strength of this association was assessed using a Cramer's V statistic.

In ants, the prevalence of *Wolbachia* in the sterile worker caste, which corresponds to a dead-end host, may vary from one nest to another [37]. We therefore specifically estimated the prevalence of *Wolbachia* within nests by screening 160 additional workers originating from eight distinct nests from both the native and the introduced range (i.e. 20 workers per nests). Half these nests were known to be sexual and the other half to be clonal. The four sexual nests originated from two populations established in the primary forest within the native range: one from French Guiana (M7) and one from Brazil (UNA). The four clonal nests originated from four genetically distinct populations: two from the native range (P2 and IP, from French Guiana and Brazil, respectively), and two from the introduced range (CA and PL, from Australia and New Caledonia, respectively).

### Genetic Characterization of the Wolbachia Strains

A phylogenetic analysis was performed to characterize the strains of *Wolbachia* detected in *W. auropunctata* populations. To this aim, we sequenced a fragment of the *wsp* gene from 74 individuals from the 17 identified infected populations from both the native and introduced range of the species, using the previously described PCR amplification protocol. PCR products were purified and sequenced on an ABI 3730 DNA sequencer (Applied Biosystems). Electrophoregrams were checked for possible errors using the Seqscape software (Applied Biosystems). Because multiple peaks were observed at some base positions in some individual electrophoregrams, infection by multiple strains was suspected in these cases. The amplification products were hence purified (QIAquick PCR Purification Kit, QIAGEN) and ligated into a plasmid vector (pGEM-T easy vector system, Promega) and transformed into JM109 competent cells (Promega). Positively transformed cells were boiled, amplified using T7 and SP6 (primers of plasmid) and sequenced with the same primers. The plasmid DNA of six clones per individual was purified and sequenced.

Our phylogenetic analysis was performed on unique *wsp* haplotypes (five haplotypes). We also included 33 additional *wsp* sequences of *Wolbachia* strains belonging to the supergroups A and B and isolated from other Formicidae available at the *Wolbachia* MLST website (http://pubmlst.org/perl/mlstdbnet/mlstdbnet.pl?page=query&file=wo_isolates.xml). The tree was rooted using a *wsp* sequence from a *Wolbachia* strain belonging to the supergroup F according to the classification of the MLST database (i.e. infecting the wasp *Apoica pallens*). All sequences were aligned using clustalW [38]. The data set was analyzed using the neighbour-joining method with Kimura two-parameter distance measure in MEGA v. 4 [39]. Bootstrap analysis was performed with 1,000 replicates.

**Figure 2:**
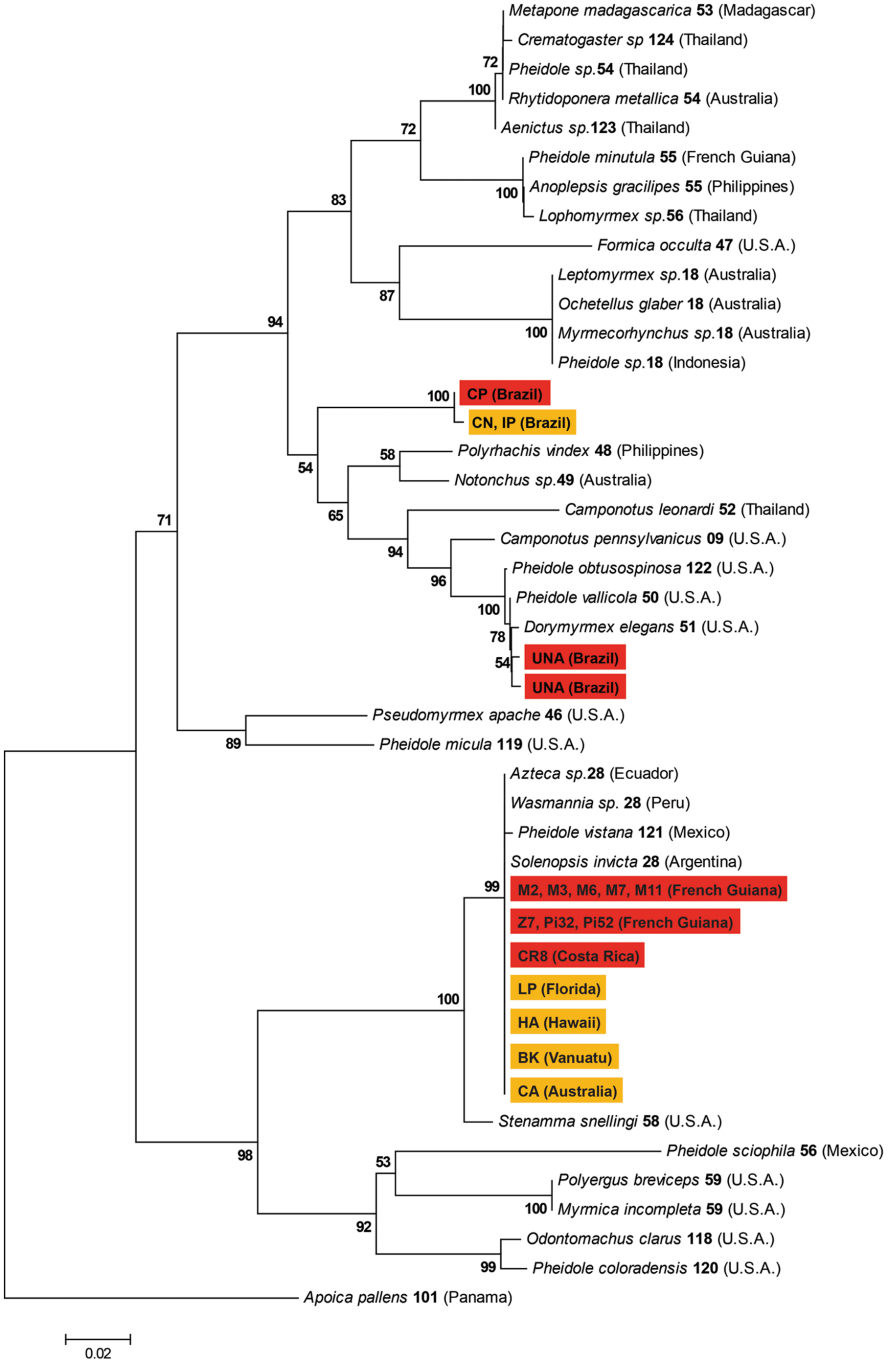
NJ tree based on the *wsp* nucleotide alignment of the different *Wolbachia* strains infecting native and introduced populations of *W. auropunctata*. Note: Each *Wolbachia* sequence is labeled with the name of its host species and the allelic number of the *wsp* sequence according to the MLST database in bold. The country of origin of each identified strain is indicated in parenthesis. Only bootstrap values (computed from 1,000 replicates) of nodes are figured for values >50%. *Wolbachia* strains found in sexual and clonal populations of *W. auropunctata* are highlighted in red and orange, respectively. Location of origin of the samples is indicated between parentheses.

Because the *wsp* gene is known to be under diversifying selection and to undergo recombination both within and between strains [40], we also conducted additional analyses on a subset of 17 individuals from the same 17 identified infected populations (one individual per population) that were analysed at the *wsp* phylogenetic analysis (see Figure 2), using the five genes used in the multilocus sequence typing approach (MLST: *gatB*, *fbpA*, *coxA*, *ftsZ* and *hcpA*). We amplified fragments of these five genes following Baldo *et al.* [41]. The PCR products were cloned and the plasmid DNA of six clones per individual were then purified and sequenced. A total of 102 sequences were thus obtained for each gene. Because of the occurrence of multiple infections, we could not conduct phylogenetic analyses on a concatenated set of MLST genes. We hence conducted five independent phylogenetic analyses following the same method as for the *wsp* gene. All haplotypes from both *wsp* and MLST genes were archived under Genbank (accession numbers JX499039–JX499070).

## Results

### Screening of Endosymbiotic Bacteria

Among the five main endosymbiotic bacteria screened in the present study (i.e. *Wolbachia*, *Cardinium*, *Arsenophonus*, *Rickettsia* and *Spiroplasma ixodetis)*, *Wolbachia* was the only one found, being present in 16 of the 42 *W. auropunctata* populations primarily screened. Together with the *Wolbachia*-specific additional screening, 18 of the 56 *W. auropunctata* populations screened were found to be infected by *Wolbachia*. We found a strong association between the reproductive system within *W. auropunctata*'s nests and the infection status, with a higher prevalence of *Wolbachia* infection in sexual than in clonal populations (*χ* ^2^ = 143.24; p – value <0.001; Cramer's V = 0.74). The mean prevalence within sexual populations was 1 and 0.90 within the queen and worker castes respectively (Table 1 and 2). In the 37 sexual nests in which queens were sampled, all 45 sexually reproducing queens as well as 138 on 142 tested workers were infected by *Wolbachia*. We found only four workers originating from two sexual nests that were *Wolbachia*-free. These workers belonged to two queenless nests from populations of French Guiana (M2, M7; Table 2). In clonal populations, the mean prevalence within populations was 0.17 and 0.15 within the queen and worker castes respectively (Table 1 and 2). We found that 122 of the 160 clonal queens (i.e. 76.25%) from the 138 sampled clonal nests were *Wolbachia*-free, irrespective to their origin (i.e. from the native or introduced range). All workers from these clonal nests were also non-infected. A total of 38 infected clonal queens were found within 23 nests of the native range in two Brazilian populations (CN and IP) and within 15 nests of the introduced range (in Hawaii, Vanuatu Islands and Australia; Table 1, Figure 1). Workers from these nests were also all infected (Table 2). Finally, five workers from a unique queenless nest originating from Florida (U.S.A) were all infected by *Wolbachia* (Table 2, Figure 1).

The prevalence of *Wolbachia* infection within nests showed a binary pattern. The 20 workers sampled from infected sexual and clonal nests were all infected. On the contrary, workers from the two *Wolbachia*-free clonal nests were all non-infected.

### Genetic Characterization of Wolbachia Strains

The *Wolbachia* strains identified in *W. auropunctata* populations all belong to the supergroup A according to the MLST classification. Among the 74 surveyed ants, four (5.4%, all originating from the same sexual population UNA in Brazil) were found to be co-infected by two distinct *Wolbachia* strains based on their *wsp* haplotypes. The co-infection of theses ants was also detected at the MLST genes. All of the other 70 ants were found to be infected by a single *wsp* haplotype. Among the subset of 17 individuals analysed using the MLST genes, six ants, all originating from different sexual populations (M2, Pi32, PI52, M2 in French Guiana and UNA and CP in Brazil) were found to be co-infected by different *Wolbachia* strains differing in at least one MLST gene. All of the analysed ants from clonal populations were found to be infected by a single *Wolbachia* strain as revealed by the genetic analyses based on both the *wsp* and MLST genes.

Examination of the *wsp* and the five MLST genes revealed the presence of 5 distinct *Wolbachia* strains, clustering in three different clades (Figure 2; Figures S1, S2, S3, S4, S5). There is no clear partitioning of this diversity between sexual and clonal populations: the most common strain is shared by both sexual and clonal populations, three other strains were specific to sexual populations, and the last strain to a clonal population. In the native range, the two infected Brazilian clonal populations (CN and IP) host a *Wolbachia* strain closely related to the one infecting the Brazilian sexual population (CP; Figure 2; Figures S1, S2, S3, S4, S5). Additionally, all infected clonal populations from the introduced range (Hawaiian, Australian, Vanuatu and some Floridian populations) harbor the same strain infecting the native French Guianese sexual populations (Figure 2; Figures S1, S2, S3, S4, S5). This last *Wolbachia* strain is identical to the one previously found to infect other New World ants [37], including *S. invicta* (Figure 2).

## Discussion

Among the five studied endosymbiotic bacteria (i.e. *Wolbachia*, *Rickettsia*, *Cardinium*, *Arsenophonus* and *Spiroplasma ixodetis*), only *Wolbachia* was detected in *W. auropunctata*. This result is not surprising given that *Wolbachia* is estimated to infect more than one third of ant species [37], while the four other endosymbionts were sporadically found in few arthropods orders [25] and seldom in ants (but see [42]–[43]). *Wolbachia* is not present in individuals (queens and workers) from most of the clonal populations, but when found, highly similar *Wolbachia* strains were also found to infect sexual populations. These results strongly suggest that the peculiar reproductive system of reproductives from *W. auropunctata* clonal populations (i.e. queen thelytokous parthenogenesis associated to male clonality) is not induced by *Wolbachia*, nor by any of the four other endosymbiotic bacteria screened in the present study.

At first glance, our study might seem to indicate an association between the reproductive system of *W. auropunctata* and the occurrence of *Wolbachia*. While individuals from clonal populations are mostly *Wolbachia*-free, all sampled queens and most of workers from sexual populations are infected by the bacterium. This pattern would suggest that *Wolbachia* infection is well established, if not favored, in sexual populations. However, identical (or almost identical) *Wolbachia* strains were found in sexual and clonal populations (Figure 2; Figures S1, S2, S3, S4, S5), a pattern in strong opposition to any direct relationship between reproductive systems and *Wolbachia* phenotypic effects. Additionally, none of the described mechanisms by which *Wolbachia* alters reproductive systems to enhance its own transmission (i.e. male killing, feminization, cytoplasmic incompatibility and obligate for oogenesis), can explain the maintenance of the bacterium in sexual populations better than in clonal populations. First, under the male killing and the feminization processes, a strong reduction of infected males is expected. Yet, we found some infected males in both native and introduced populations of *W. auropunctata* (data not shown). Additionally, feminization through known *Wolbachia* mediated-mechanisms would produce sterile haploid females, rendering this phenotype unlikely within the ants [21]–[44]. Second, one could argue that *Wolbachia* could be obligatory for oogenesis in *W. auropunctata*, as was found in the hymenopteran genus *Asobara* [45]. However, in the case of *W. auropunctata*, *Wolbachia*-free clonal queens use sexual reproduction to produce workers and *Wolbachia*-infected clonal queens produce parthenogenetic daughters, two features arguing against a role of *Wolbachia* in oogenesis in this species. Finally, cytoplasmic incompatibility, the most prevalent phenotypic effect induced by *Wolbachia* [22], is unlikely to be differentially maintained in sexual vs. clonal in *W. auropunctata* because in both types of populations workers are produced sexually. Parthenogenetic queens like queens from sexual populations may hence suffer from CI through their workers produced sexually.

The *Wolbachia* infection pattern seems rather associated to the invasive status of *W. auropunctata* populations, even if this pattern is mainly driven by French Guianese samples due to the sampling scheme. The loss of endosymbiotic bacteria in invasive populations is common in ant species [18]–[20]. Two main hypotheses were proposed to explain this loss: (i) *Wolbachia* can be eliminated through drift during introduction if all founders were uninfected [18], or (ii) *Wolbachia* can be lost in invasive populations after the introduction through drift or selection [18]–[19]. In the case of *W. auropunctata*, drift seems unlikely to account for the results because clonal populations have recurrently emerged from sexual populations [30] and the transmission of *Wolbachia* in sexual populations was found to be nearly perfect. It is therefore unlikely that *Wolbachia* could have been lost in multiple invasive clonal populations through the sole means of drift. Interestingly, the emergence of invasiveness in *W. auropunctata* follows an important habitat change [27]. Two non-exclusive alternative hypotheses based on ecological features relative to this habitat change might hence explain the infection pattern observed in *W. auropunctata* populations.

First, clonal populations may have passively lost *Wolbachia* by natural heat treatment. While non-invasive sexual populations are established in primary forests with low temperature variation below 30°C, clonal populations settle in human-modified areas characterized by hotter and drier microclimates (reaching as far as 40°C; [28]). Additional work by our group demonstrated that workers from clonal populations tolerate temperatures as high as 36°C, while the mortality rate in workers from sexual populations reach 40% at this temperature (unpublished data). *Wolbachia* is known to be sensitive to such temperatures and heat treatments are commonly used to remove it from hosts for experimental purposes [46]. Classically, these treatments require rearing hosts’ larvae at 33°C to 35°C for few days to several generations. It is therefore possible that the abiotic conditions of human-modified areas lead to a loss of *Wolbachia* infection in these zones, i.e. in introduced and native clonal populations of *W. auropunctata*.

A second hypothesis is that *Wolbachia* has been actively lost in clonal populations from human-modified habitats through relaxed selection and/or counter-selection against infected individuals. *Wolbachia* has been shown to induce both costs (e.g. reduction of fecundity, adult survival and locomotor performance; [4]–[5]) and benefits (e.g., protection from RNA viruses, upregulation of immunity-gene expression against *Plasmodium* and filarial nematodes; [7]–[47]) for infected individuals. In the case of *W. auropunctata*, sexual populations established in primary forests are likely to face more important biotic pressures than clonal populations established in human-modified areas, notably through higher levels of interspecific interactions with other ant species [28]. The fitness cost-benefit ratio might hence favor the loss of *Wolbachia* in human-modified areas, and its maintenance in primary forests.

Both hypotheses could explain the unique case of infected native clonal populations (i.e., CN and IP in Brazil; Table 1, 2). The habitats of these particular populations correspond to recently abandoned shaded cocoa plantations where there has been no human activity for at least 10 years. In these exploitations (i.e. shaded cocoa plantations), the vegetation structure and stratification are considered to be similar to, albeit less complex than, that of natural forests. The ant species richness in these plots roughly corresponds to that of a native forest of low diversity [48]. During exploitation both biotic and abiotic environmental conditions, hence, were different from those of typical habitat where clonal populations are found. Furthermore, since the cessation of human activities, vegetation has grown back so as to change the environmental conditions at the ground level, in particular in reducing the daily and seasonal thermal amplitudes. Under these conditions that enable complex biotic interactions (including higher levels of parasitism), *Wolbachia* could have been maintained in *W. auropunctata* clonal populations established on these sites, due to a possible beneficial role against pathogens. This result strengthens the apparent close association between environmental parameters and *Wolbachia* infection in *W. auropunctata*.

Finally, the occurrence of *Wolbachia* in clonal invasive populations outside the native range, in Florida, Hawaii, Australia, and in the Vanuatu Islands, cannot be explained by the two above hypotheses based on ecological features. Interestingly however, the infection of these populations is consistent with their invasion history. Populations established in Florida, Hawaii, Australia, and in the Vanuatu Islands were found to be infected by the same *Wolbachia* strain. These populations have previously been shown to share a unique mitochondrial haplotype and to display closely related clonal queens genotypes at microsatellite markers [35]–[49]. The distribution and identity of the *Wolbachia* strain uncovered in the infected introduced populations are therefore consistent with previous studies. Consequently, these invasive populations are likely to originate from the same ancestral clonal native population and the infection by *Wolbachia* most probably occurred once in this ancestral population before long dispersal events. This ancestral population remains, however, unknown.

In conclusion, this study revealed that, except *Wolbachia,* none of the reproductive parasite screened in the present study infect wild populations of *W. auropunctata*. Furthermore, presence or absence of *Wolbachia* infection is unlikely to explain the reproductive system polymorphism found in *W. auropunctata*. The infection pattern of *Wolbachia* in *W. auropunctata* rather echoes with previous studies illustrating a loss of *Wolbachia* in invasive populations [18]–[20]. The most likely explanation is that this loss resulted from natural heat treatment and/or relaxed selection during a shift in habitat in invasive populations. Putative immunological benefits and/or physiological costs induced by *Wolbachia* should be experimentally tested in the future to distinguish between these hypotheses.

## Supporting Information

Figure S1 **NJ tree based on the** ***gatB*** **nucleotide alignment of the different Wolbachia strains infecting native and introduced populations of** ***W. auropunctata*** **.** Note: *Wolbachia* strains found in sexual and clonal populations of *W. auropunctata* are highlighted in red and orange, respectively. Each other *Wolbachia* sequence is labeled with the name of its host species and its respective GenBank Accession number in bold. Location of origin of the samples is indicated between parentheses. Only bootstrap values (computed from 1,000 replicates) of nodes are figured for values >50%.
(DOC)

Figure S2 **NJ tree based on the** ***fbpA*** **nucleotide alignment of the different Wolbachia strains infecting native and introduced populations of** ***W. auropunctata*** **.** Note: *Wolbachia* strains found in sexual and clonal populations of *W. auropunctata* are highlighted in red and orange, respectively. Each other *Wolbachia* sequence is labeled with the name of its host species and its respective GenBank Accession number in bold. Location of origin of the samples is indicated between parentheses. Only bootstrap values (computed from 1,000 replicates) of nodes are figured for values >50%.
(DOC)

Figure S3 **NJ tree based on the** ***CoxA*** **nucleotide alignment of the different Wolbachia strains infecting native and introduced populations of** ***W. auropunctata*** **.** Note: *Wolbachia* strains found in sexual and clonal populations of W. auropunctata are highlighted in red and orange, respectively. Each other Wolbachia sequence is labeled with the name of its host species and its respective GenBank Accession number in bold. Location of origin of the samples is indicated between parentheses. Only bootstrap values (computed from 1,000 replicates) of nodes are figured for values >50%.
(DOC)

Figure S4 **NJ tree based on the** ***ftsZ*** **nucleotide alignment of the different Wolbachia strains infecting native and introduced populations of** ***W. auropunctata*** **.** Note: *Wolbachia* strains found in sexual and clonal populations of *W. auropunctata* are highlighted in red and orange, respectively. Each other *Wolbachia* sequence is labeled with the name of its host species and its respective GenBank Accession number in bold. Location of origin of the samples is indicated between parentheses. Only bootstrap values (computed from 1,000 replicates) of nodes are figured for values >50%.
(DOC)

Figure S5 **NJ tree based on the** ***hcpA*** **nucleotide alignment of the different Wolbachia strains infecting native and introduced populations of** ***W. auropunctata*** **.** Note: *Wolbachia* strains found in sexual and clonal populations of *W. auropunctata* are highlighted in red and orange, respectively. Each other *Wolbachia* sequence is labeled with the name of its host species and its respective GenBank Accession number in bold. Location of origin of the samples is indicated between parentheses. Only bootstrap values (computed from 1,000 replicates) of nodes are figured for values >50%.
(DOC)
