# Supplementary material for: Distribution of Endosymbiotic Reproductive Manipulators Reflects Invasion Process and Not Reproductive System Polymorphism in the Little Fire Ant Wasmannia auropunctata
Source: PLoS One. 2013 Mar 11;8(3):e58467. doi: 10.1371/journal.pone.0058467 (PMC3594316; doi:10.1371/journal.pone.0058467)

**Figure S4:** **NJ tree based on the f*tsZ* nucleotide alignment of the different *Wolbachia* strains infecting native and introduced populations of *W. auropunctata*.**

Note: *Wolbachia* strains found in sexual and clonal populations of *W. auropunctata* are highlighted in red and orange, respectively. Each other *Wolbachia* sequence is labeled with the name of its host species and its respective GenBank Accession number in bold. Location of origin of the samples is indicated between parentheses. Only bootstrap values (computed from 1,000 replicates) of nodes are figured for values > 50%.


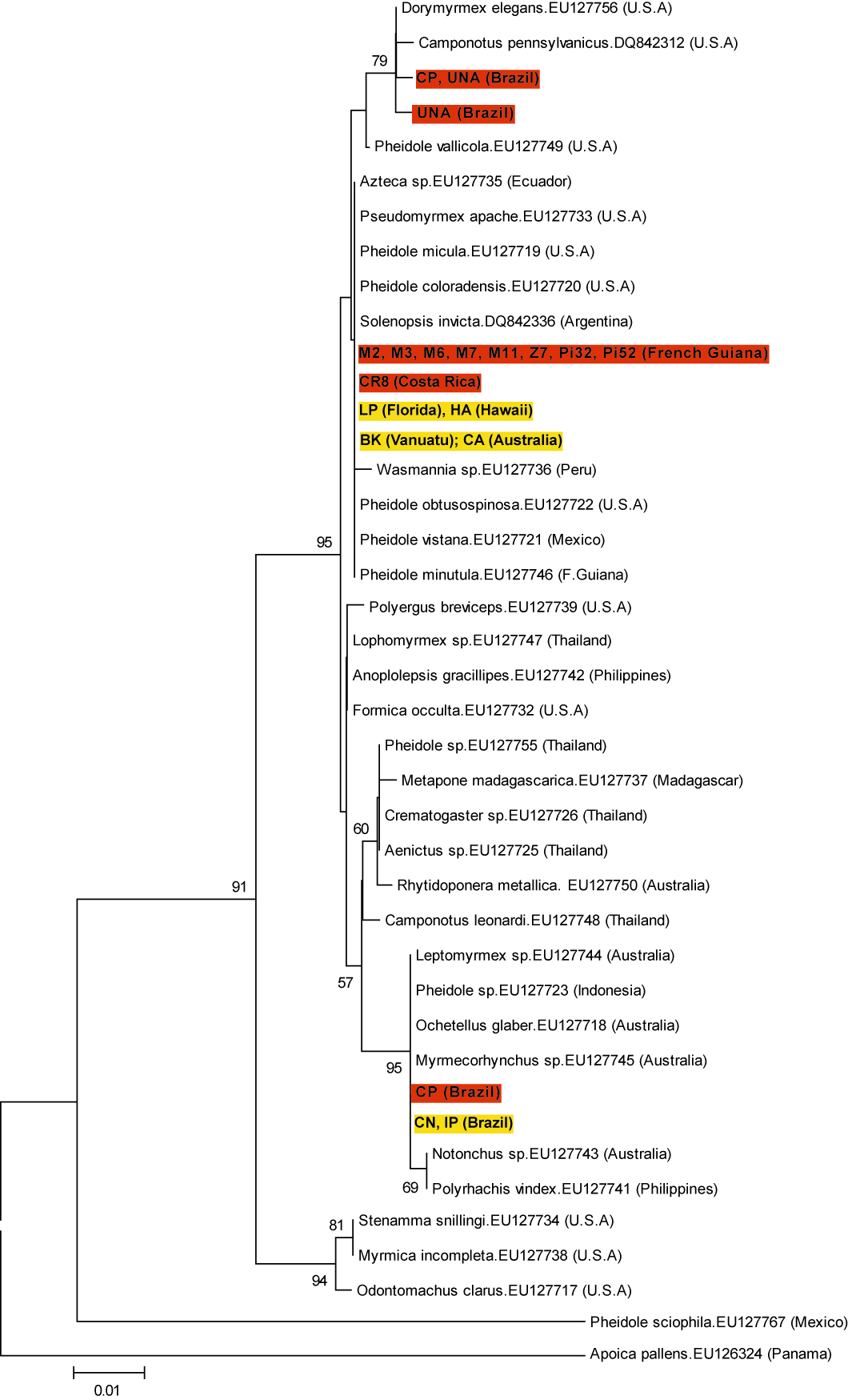

Supplement: Figure S4 — NJ tree based on the ftsZ nucleotide alignment of the different Wolbachia strains infecting native and introduced populations of W. auropunctata . Note: Wolbachia strains found in sexual and clonal populations of W. auropunctata are highlighted in red and orange, respectively. Each other Wolbachia sequence is labeled with the name of its host species and its respective GenBank Accession number in bold. Location of origin of the samples is indicated between parentheses. Only bootstrap values (computed from 1,000 replicates) of nodes are figured for values >50%. (DOC) [file pone.0058467.s004.doc]
